# Effect of short-term oral prednisone therapy on blood gene expression: a randomised controlled clinical trial

**DOI:** 10.1186/s12931-019-1147-2

**Published:** 2019-08-05

**Authors:** Hiroto Takiguchi, Virginia Chen, Ma’en Obeidat, Zsuzsanna Hollander, J. Mark FitzGerald, Bruce M. McManus, Raymond T. Ng, Don D. Sin

**Affiliations:** 1 The University of British Columbia Centre for Heart Lung Innovation (HLI), St Paul’s Hospital, 1081 Burrard Street, Vancouver, BC V6Z 1Y6 Canada; 2 Prevention of Organ Failure (PROOF) Centre of Excellence, 10th floor, 1190 Hornby Street, Vancouver, BC V6Z 2K5 Canada; 3 Respiratory Division, Department of Medicine, Gordon and Leslie Diamond Health Care Centre, University of British Columbia, 7th Floor, 2775 Laurel Street, Vancouver, BC V5Z 1M9 Canada; 4 Division of Pulmonary Medicine, Department of Medicine, Tokai University School of Medicine, 143 Shimokasuya, Isehara, Kanagawa 259-1193 Japan; 5 Department of Computer Science, University of British Columbia, ICICS/CS Building 201-2366 Main Mall, Vancouver, BC V6T 1Z4 Canada

**Keywords:** Chronic obstructive pulmonary disease, Acute exacerbation, Blood, Prednisone, Gene expression, Microarray, Mortality

## Abstract

**Background:**

Effects of systemic corticosteroids on blood gene expression are largely unknown. This study determined gene expression signature associated with short-term oral prednisone therapy in patients with chronic obstructive pulmonary disease (COPD) and its relationship to 1-year mortality following an acute exacerbation of COPD (AECOPD).

**Methods:**

Gene expression in whole blood was profiled using the Affymetrix Human Gene 1.1 ST microarray chips from two cohorts: 1) a prednisone cohort with 37 stable COPD patients randomly assigned to prednisone 30 mg/d + standard therapy for 4 days or standard therapy alone and 2) the Rapid Transition Program (RTP) cohort with 218 COPD patients who experienced AECOPD and were treated with systemic corticosteroids. All gene expression data were adjusted for the total number of white blood cells and their differential cell counts.

**Results:**

In the prednisone cohort, 51 genes were differentially expressed between prednisone and standard therapy group at a false discovery rate of < 0.05. The top 3 genes with the largest fold-changes were KLRF1, GZMH and ADGRG1; and 21 genes were significantly enriched in immune system pathways including the natural killer cell mediated cytotoxicity. In the RTP cohort, 27 patients (12.4%) died within 1 year after hospitalisation of AECOPD; 32 of 51 genes differentially expressed in the prednisone cohort significantly changed from AECOPD to the convalescent state and were enriched in similar cellular immune pathways to that in the prednisone cohort. Of these, 10 genes including CX3CR1, KLRD1, S1PR5 and PRF1 were significantly associated with 1-year mortality.

**Conclusions:**

Short-term daily prednisone therapy produces a distinct blood gene signature that may be used to determine and monitor treatment responses to prednisone in COPD patients during AECOPD.

**Trial registration:**

The prednisone cohort was registered at clinicalTrials.gov (NCT02534402) and the RTP cohort was registered at ClinicalTrials.gov (NCT02050022).

**Electronic supplementary material:**

The online version of this article (10.1186/s12931-019-1147-2) contains supplementary material, which is available to authorized users.

## Background

Chronic obstructive pulmonary disease (COPD) is a leading cause of morbidity and mortality in the United States (US) and elsewhere. In the US, COPD is now the 3rd leading cause of morbidity and mortality, trailing only ischemic heart disease and lung cancer [1]. Most COPD deaths occur during periods of acute exacerbations (AECOPDs), which are characterised by an abrupt increase in dyspnea and cough related most commonly to an acute respiratory tract infection. AECOPDs are common indications for hospitalisation. In the US each year, there are more than 225,000 hospitalisations related to AECOPD [2]. Most national and international guidelines and strategic documents recommend the use of systemic corticosteroids with or without antibiotics for serious AECOPD. Although on average systemic corticosteroids reduce the length of stay in hospital (by approximately 1 day) and decrease the risk of treatment failure, there is tremendous variation in response across patients with some experiencing benefits while others experiencing only harm [3]. Moreover, even with “successful” therapy of their AECOPDs, a third of patients treated with systemic corticosteroids experience a treatment relapse (i.e. re-exacerbation) within 6 months [4] and 10–30% die within 1 year of their major AECOPD [5, 6]. The use of systemic corticosteroids during AECOPD is also associated with adverse events including hyperglycemia (which impacts ~ 50% of patients), hypertension (which impacts ~ 15% of patients) and adrenal insufficiency (which impacts 2 to 10% of patients) [3, 4]. Despite these challenges and limitations of systemic corticosteroids, they have been the mainstay of AECOPD management over the past 30 years. In the era of Precision Medicine, it is essential that we identify easily accessible biomarkers that can guide the use of systemic corticosteroids to patients who will benefit the most from these therapies. Here, we report on findings from 2 studies: 1) a clinical trial that determined which genes in peripheral blood were most responsive to short-term oral prednisone therapy in patients with COPD (i.e., discovery of blood biomarkers related to prednisone therapy) and 2) a clinical study which related the most responsive genes from the clinical trial to 1 year mortality in patients who experienced a serious AECOPD and were treated with systemic corticosteroids in hospital (i.e., discovery of predictive biomarkers of prednisone). Through these combined studies, we sought to identify genetic biomarkers that could be used to predict therapeutic benefits of systemic corticosteroids during AECOPD.

## Methods

### Study 1: a clinical trial to determine which genes in peripheral blood of COPD patients are responsive to oral prednisone therapy (NCT02534402)

To determine which genes were responsive to oral prednisone therapy in patients with COPD, we performed a randomised clinical trial (NCT02534402). We enrolled 40 patients from the COPD Clinic at St. Paul’s Hospital (SPH) in Vancouver, Canada. All patients had a clinical diagnosis of COPD from a board-certified pulmonologist and demonstrated a forced expiratory volume in 1 s (FEV1) to forced vital capacity (FVC) ratio of less than 70% (post-bronchodilator). The other enrollment criteria were that participants had to be free of exposure to prednisone or any other systemic corticosteroids and clinically stable (i.e., free of AECOPD) for at least 2 weeks prior to study entry. Following informed consent, patients were randomised in a 3: 1 ratio to either prednisone (30 mg/d for 4 days) + standard therapy or standard therapy alone. Participants attended 5 study visits over 5 consecutive days. The prednisone group received 30 mg/d of prednisone from days 1 through 4. Compliance was directly observed by the study personnel. During the trial period, patients were not permitted to modify any of their inhaled therapies including the dosage of inhaled corticosteroids or be placed on oral antibiotics.

During each of the visits, blood samples were collected in PAXgene (PreAnalytix, Switzerland) and EDTA tubes just before dispensation of the oral prednisone tablets. The PAXgene samples were placed in a -80C freezer. The EDTA blood was spun down within 2 h of collection and plasma and buffy coat were aliquoted and stored at -80C until analysis. Total RNA was extracted from PAXgene tubes using a PAXgene Blood miRNA kit from PreAnalytix, according to the manufacturer’s instructions; mRNA abundance was measured using Human Gene 1.1 ST 96-well array plates (Affymetrix, United States) at the McGill University and Genome Quebec Innovation Centre.

### Study 2: rapid transition program cohort (NCT02050022)

To determine which if any of the steroid-responsive genes discovered in study 1 could be replicated and which of these replicated genes related to 1-year mortality among patients who experienced a severe AECOPD, requiring hospitalisation, we used data from the Rapid Transition Program (RTP) cohort. The details of the RTP cohort have been previously reported [7]. Briefly, RTP included patients admitted for AECOPD as the Principal (or Most Responsible) Diagnosis at Vancouver General Hospital (VGH) or St. Paul’s Hospital (SPH) in Vancouver, Canada. The diagnosis of AECOPD was made based on clinical judgement by board-certified pulmonologists or general internists who were the most responsible physicians for these patients, and later confirmed by an independent board-certified pulmonologist, who did not participate in the care of these patients. During the hospitalisation, all patients were treated with at least 4 days of systemic corticosteroids (at the discretion of the attending physicians). Blood samples for this study were collected at baseline (within first 3 days of hospitalization for AECOPD) and between day 30 and 90 post-hospitalisation when the patients were completely stable and their symptoms had returned to baseline levels (i.e., convalescent sample). Similar to study 1, all blood samples were collected in PAXgene and EDTA tubes and were stored at -80C until processing. CBC and differential were performed on all samples collected in EDTA tubes.

### Statistical analysis

Statistical analyses were performed in R. Patient characteristics were assessed for statistical significance using a Student’s *t*-test and a Kruskal-Wallis test for continuous variables, and a Fisher’s exact test for categorical variables. The *oligo* package was used to assess the quality of CEL files based on the normalized unscaled standard error (NUSE) and relative log-expression (RLE) metrics, and perform normalization using Robust Multi-array Average. Probes were summarized at the transcript cluster level. The ComBat algorithm was used to correct for plate-to-plate technical variation. The resulting batch-corrected, transcript cluster-level data was annotated with Human Gene 1.1 ST transcript cluster annotations (v36). Transcripts that were not annotated or mapped to more than one gene, were removed. If a gene had multiple transcripts assigned to it, these transcripts were averaged into a single value per sample. The end result of these steps is referred to as the gene expression data.

Diffearential expression was assessed using the *limma* package, which performs a moderated t-test using empirical Bayes. All analyses were adjusted for white blood cell counts and percentage of lymphocytes, neutrophils, monocytes, basophils, and eosinophils and were blocked by subject (i.e., they were modeled as random effects). Genes with a false discovery rate (FDR) < 0.05 between prednisone-treated versus untreated samples were considered differentially expressed (“prednisone genes”). These were then tested for functional enrichment analysis using the web-based gene set analysis toolkit (WebGestalt) [8]. Gene symbols were used as input for WebGestalt and tested for enrichment in Gene Ontology (GO) processes [9] and Kyoto Encyclopedia of Genes and Genomes (KEGG) pathways [10]. Enrichment was analyzed by a hypergeometric model and adjusted for multiple testing, with a FDR < 0.05 being considered significant.

The prednisone genes were replicated in the RTP cohort by comparing their convalescent gene expression to baseline AECOPD gene expression values. A FDR < 0.05 was considered significant in the replication dataset. The replicated genes were then used in a time-to-event analysis, to determine if any of these were predictive of mortality in the RTP cohort. Baseline AECOPD gene expression was subtracted from convalescent gene expression levels (to obtain “deltas”, or changes in these genes) on a log 2 scale, corresponding to a ratio on the intensity scale. These deltas were tested for their ability to predict mortality in a time-to-event analysis, for each gene individually. This was performed by fitting Cox proportional-hazards models (*survival* package) to predict number of days between AECOPD and death. All analyses were adjusted for white blood cell counts and percentage of lymphocytes, neutrophils, monocytes, basophils, and eosinophils. Genes with a *p*-value < 0.05 were considered significant in this time-to-event analysis (“mortality genes”). These mortality genes were combined together in a single Cox proportional-hazards mode.

## Results

### Study 1: a clinical trial to determine which genes in peripheral blood of COPD patients are responsive to oral prednisone therapy

**Fig. 1:**
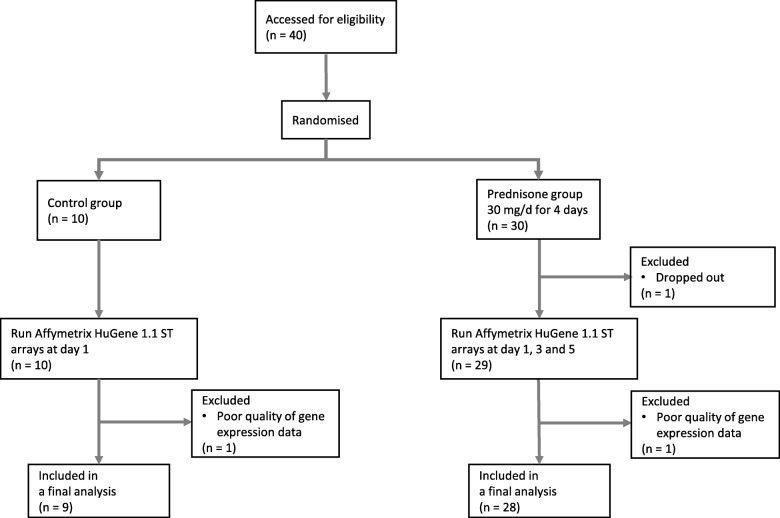
A flow diagram of participants. In study 1, 40 patients were enrolled and were randomised to either prednisone (30 mg/d for 4 days) or control group in a 3: 1 ratio. Participants attended 5 study visits over 5 consecutive days; 28 and 9 patients were included in the final analysis after excluding 2 and 1 patients from prednisone and control groups, respectively

A flow diagram of patient recruitment is shown in Fig. 1; 30 patients were randomised to the prednisone arm and 10 were allocated to the control group. After excluding 2 patients in the prednisone group and 1 patient in the control group because of incomplete sample data collection or poor microarray data quality, there were 28 in the prednisone and 9 patients in the control group in the final analysis. The demographic and clinical data of the cohort stratified by prednisone treatment status are summarized in Table 1. There were no significant differences between the two groups in terms of age, gender, ethnicity, smoking status, a history of asthma or complete blood count (CBC) including its differential white blood cell count. However, patients in the prednisone group had a lower FEV_1_, % predicted (*p* = 0.007), FVC, % predicted (*p* = 0.020) and were slightly more likely to have used inhaled corticosteroids at study entry (*p* = 0.041).

**Table 1:** Baseline characteristics of patients in study 1 and study 2

	Study 1			Study 2
	Control group *n* = 9	Prednisone group *n* = 28	*P-*value	*n* = 218
Age, years	61.7 ± 10.8	64.0 ± 9.8	0.543	67.9 ± 11.6
Male	5 (55.6)	22 (78.6)	0.215	137 (62.8)
Ethnicity			0.665	
Caucasian	9 (100)	24 (85.7)		173 (79.4)
First nations	0 (0)	3 (10.7)		24 (11.1)
African	0 (0)	1 (3.6)		1 (0.5)
Others	0 (0)	0 (0)		20 (9.2)
Smoking status			0.462	
Former	3 (33.3)	14 (50)		84 (38.5)
Current	6 (66.7)	14 (50)		122 (56.0)
Pack-year smoked	28.2 ± 22.3 (*n* = 5)	33.7 ± 39.3 (*n* = 16)	0.771	12 (5.5)
Pulmonary function test				
FEV_1_/FVC, %	60.2 ± 9.7 (*n* = 6)	52.1 ± 15.1 (*n* = 21)	0.227	56.2 ± 15.7 (*n* = 78)
FEV_1_, % predicted	78.4 ± 12.1 (*n* = 7)	53.3 ± 21.8 (*n* = 23)	0.007	55.0 ± 21.3 (*n* = 102)
FVC, % predicted	104 ± 18 (*n* = 7)	83.7 ± 18.5 (*n* = 21)	0.020	76.3 ± 22.3 (*n* = 78)
GOLD grades	*n* = 7	*n* = 23	0.096	*n* = 102
1 or 2	4 (57.1)/3 (42.9)	3 (13.0)/11 (47.8)		15 (14.7)/47 (46.1)
3 or 4	0 (0)/0 (0)	5 (21.7)/4 (17.4)		24 (23.5)/16 (15.7)
Asthma	3 (33.3)	9 (32.1)	1.000	52 (24.1) (*n* = 216)
Laboratory measurements				
White blood cells (× 10^3^/μL)	6.48 ± 1.11	7.21 ± 1.95	0.296	9.58 ± 4.76 (*n* = 121)
Neutrophil, %	63.5 ± 5.0	62.5 ± 10.3	0.620	79.2 ± 14.1 (*n* = 120)
Monocyte, %	5.99 ± 1.21	5.90 ± 1.44	0.790	6.09 ± 3.82 (*n* = 120)
Eosinophil, %	2.94 ± 1.34	4.13 ± 4.48	0.832	0.87 ± 1.36 (*n* = 120)
Basophil, %	0.56 ± 0.26	0.61 ± 0.25	0.484	1.74 ± 7.29 (*n* = 120)
Lymphocyte, %	24.5 ± 5.0	24.6 ± 8.2	0.818	11.9 ± 8.8 (*n* = 120)
Pharmacotherapy				
Bronchodilator	8 (88.9)	24 (85.7)	1.000	216 (99.1)
Inhaled corticosteroid	4 (44.4)	23 (82.1)	0.041	163 (74.8)

Values are means ± SD or numbers (%) of observations

GOLD grade was defined as grade 1: FEV_1_ ≥ 80% predicted; grade 2: FEV_1_ 50–79% predicted; grade 3: FEV_1_ 30–49% predicted; grade 4: FEV_1_ < 30% predicted.

*FEV1* forced expiratory volume in 1 s, *FVC* forced vital capacity, *GOLD* the Global Initiative for Chronic Obstructive Lung Disease

**Fig. 2:**
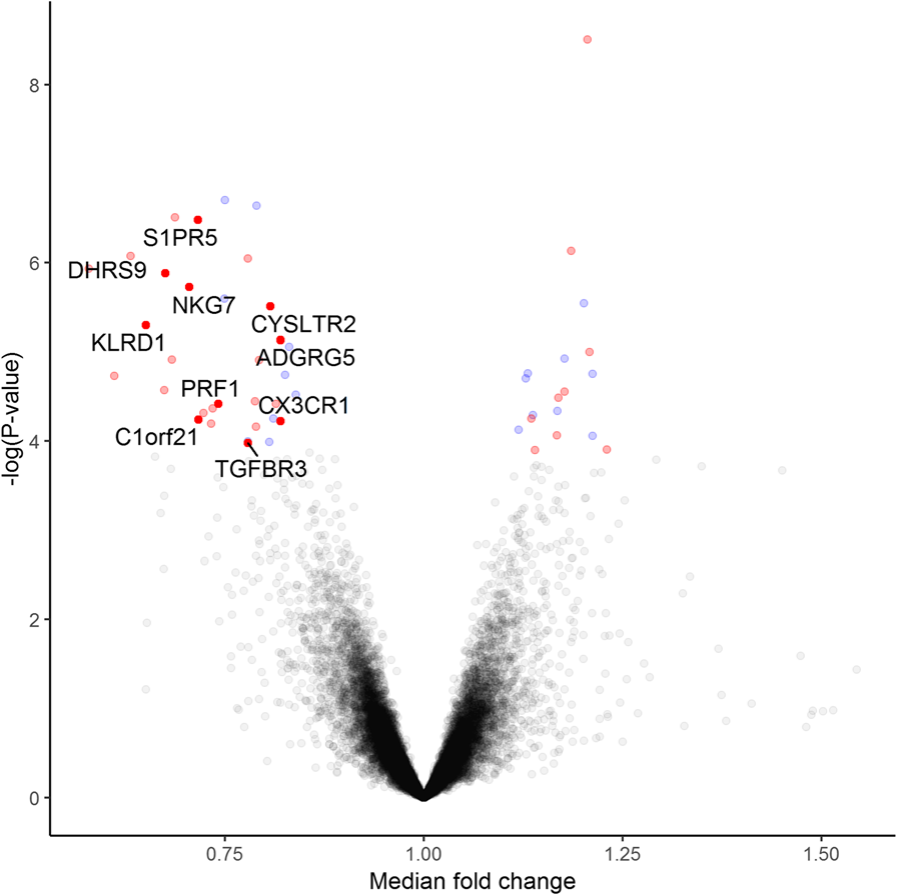
A Volcano plot of gene differentially expressed between prednisone and control group. The plot shows the fold-change on the X-axis versus the unadjusted *P* values (on the–log10 scale) on the Y-axis. Genes at a FDR < 0.05 for differential expression in study 1 are annotated on the graph (coloured dots). The red dots represent replicated genes in study 2 and genes that related to 1-year mortality are represented as dark red dots

There was no significant differences in the (baseline) day 1 transcriptomic expression of any of the measured genes at a FDR < 0.05 between the prednisone and control groups. However, in the prednisone arm, at day 3 and 5, there were 12 and 17 genes, respectively, that were differentially expressed at a FDR < 0.05 when compared with day 1, after adjusting for the total number of white blood cells and its differential cell count (Additional file 1: Table S1 and Additional file 2: Table S2). There were no genes that were differentially expressed between day 3 and 5 among those who received daily prednisone therapy. Therefore, for the remaining analysis, we pooled the gene expression from these two timepoints together to increase statistical power. When we compared days 3 and 5 from the prednisone group to day 1 from both the prednisone and control groups, there were 857 genes were differentially expressed at a FDR < 0.05 without adjusting for total number of white blood cells and their differential cell count, and only 51 genes with adjustments (Fig. 2).

Among these 51 differently expressed genes in the adjusted analysis at a FDR < 0.05, 33 genes were down-regulated and 18 genes were up-regulated. The gene with the greatest magnitude of fold-change was killer cell lectin-like receptor subfamily F, member 1 (KLRF1; fold change [FC], 1.73). The gene with the second greatest fold-change was granzyme H (GZMH; FC, 1.64), followed by adhesion G protein-coupled receptor G1 (ADGRG1; FC, 1.58) and killer cell lectin-like receptor subfamily D, member 1 (KLRD1, FC; 1.54) (Table 2). All 51 genes are shown in Additional file 3: Table S3.

**Table 2:** The top 30 genes differentially expressed by prednisone at 5% FDR in study 1

Gene	GeneName	FC	*P*-value	FDR	Direction
KLRF1	killer cell lectin-like receptor subfamily F, member 1	1.73	1.16E-06	2.48.E-03	down
GZMH	granzyme H	1.64	1.84E-05	1.42.E-02	down
ADGRG1	adhesion G protein-coupled receptor G1	1.58	8.37E-07	2.14.E-03	down
KLRD1	killer cell lectin-like receptor subfamily D, member 1	1.54	5.04E-06	6.46.E-03	down
GZMA	granzyme A	1.48	2.68E-05	1.90.E-02	down
DHRS9	dehydrogenase/reductase (SDR family) member 9	1.48	1.31E-06	2.52.E-03	down
PDGFD	platelet derived growth factor D	1.46	1.22E-05	1.13.E-02	down
GZMB	granzyme B	1.46	3.08E-07	1.27.E-03	down
NKG7	natural killer cell granule protein 7	1.42	1.88E-06	3.29.E-03	down
S1PR5	sphingosine-1-phosphate receptor 5	1.40	3.30E-07	1.27.E-03	down
C1orf21	chromosome 1 open reading frame 21	1.40	5.70E-05	2.74.E-02	down
GNLY	granulysin	1.38	4.84E-05	2.59.E-02	down
WLS	wntless Wnt ligand secretion mediator	1.36	6.34E-05	2.91.E-02	down
SYTL2	synaptotagmin-like 2	1.36	4.29E-05	2.43.E-02	down
PRF1	perforin 1 (pore forming protein)	1.35	3.83E-05	2.23.E-02	down
SLAMF7	SLAM family member 7	1.33	2.53E-06	4.06.E-03	down
SPON2	spondin 2, extracellular matrix protein	1.33	1.96E-07	1.27.E-03	down
TGFBR3	transforming growth factor beta receptor III	1.28	1.05E-04	4.11.E-02	down
NCR1	natural cytotoxicity triggering receptor 1	1.28	1.00E-04	4.09.E-02	down
PIGB	phosphatidylinositol glycan anchor biosynthesis class B	1.28	8.89E-07	2.14.E-03	down
FAM174A	family with sequence similarity 174, member A	1.27	3.55E-05	2.20.E-02	down
CEP78	centrosomal protein 78 kDa	1.27	6.84E-05	3.06.E-02	down
ID2	inhibitor of DNA binding 2, dominant negative helix-loop-helix protein	1.27	2.27E-07	1.27.E-03	down
ABCB1	ATP binding cassette subfamily B member 1	1.26	1.23E-05	1.13.E-02	down
OSBPL5	oxysterol binding protein-like 5	1.24	1.02E-04	4.09.E-02	down
CYSLTR2	cysteinyl leukotriene receptor 2	1.24	3.08E-06	4.23.E-03	down
SMPDL3A	sphingomyelin phosphodiesterase, acid-like 3A	1.23	5.57E-05	2.74.E-02	down
MILR1	mast cell immunoglobulin-like receptor 1	1.23	1.24E-04	4.78.E-02	up
USP28	ubiquitin specific peptidase 28	1.23	3.83E-05	2.23.E-02	down
CX3CR1	chemokine (C-X3-C motif) receptor 1	1.22	5.92E-05	2.78.E-02	down

The pooled data in prednisone group between day 3 and day 5 was compared with the pooled data in prednisone and control group at day 1. All gene expression data were adjusted for the total number of white blood cells and its differential cell count

*FDR* false discovery rate, *FC* fold change

Enrichment analysis was undertaken using prednisone genes at a FDR < 0.05 (Table 3), resulting in 21 genes that were significantly enriched in 11 GO biological processes. All of these gene were related to the immune system except one that was related to the circadian rhythm. The highest regulated GO processes were immune responses (*p* = 2.57E-07), followed by cytolysis (*p* = 1.19E-06), and cellular defense response (*p* = 2.87E-05). KEGG pathway analysis also significantly included 4 pathways; graft-versus-host disease (*p* = 1.43E-05), natural killer (NK) cell mediated cytotoxicity (*p* = 1.80E-05), neuroactive ligand-receptor interaction (*p* = 0.0037) and metabolic pathway (*p* = 0.0392). All genes in the pathway of NK cell mediated cytotoxicity including KLRD1, PRF1, GZMB and NCR1 were down-regulated by the use of prednisone (Additional file 4: Figure S1).

**Table 3:** Enrichment analysis: Biological process and pathway regulated by prednisone in study 1

Biological process and pathway	*P*-value	FDR	Genes
Immune response	2.57E-07	8.40E-05	KLRD1, TGFBR3, IKBKE, GZMA, CX3CR1, SPON2, MILR1, NCR1, SLAMF7, CYSLTR2, NFKB2, CD36, PRF1, NR1D1, PAG1
Cytolysis	1.19E-06	2.00E-04	GZMA, PRF1, GZMB, GZMH
**Graft-versus-host disease**	**1.43E-05**	**3.60E-05**	**KLRD1, PRF1, GZMB**
**Natural killer cell mediated cytotoxicity**	**1.80E-05**	**3.60E-05**	**KLRD1, PRF1, GZMB, NCR1**
Cellular defense response	2.87E-05	0.0028	PRF1, CX3CR1, GNLY, NCR1
Immune system process	3.44E-05	0.0028	KLRD1, GZMA, CX3CR1, NCR1, SLAMF7, ID2, CYSLTR2, CD36, PAG1, TGFBR3, IKBKE, MILR1, SPON2, NFKB2, NR1D1, PRF1
Circadian rhythm	0.0001	0.0065	ID2, PRF1, NR1D1, PER2
cell activation	0.0003	1.40E-02	CX3CR1, MILR1, NCR1, SLAMF7, ID2, NFKB2, CD36, STXBP1, PAG1
Natural killer cell activation	0.0003	1.40E-02	ID2, SLAMF7, NCR1
Regulation of immune system process	0.0009	3.68E-02	KLRD1, IKBKE, MILR1, NCR1, ID2, NFKB2, NR1D1, CD36, PAG1
Cell killing	0.0011	3.92E-02	SLAMF7, GNLY, NCR1
Regulation of immune response	0.0012	3.92E-02	KLRD1, IKBKE, NFKB2, CD36, NR1D1, PAG1, NCR1
Defense response	0.0014	4.16E-02	IKBKE, CX3CR1, SPON2, NCR1, SLAMF7, NFKB2, CD36, NR1D1, PRF1, GNLY
**Neuroactive ligand-receptor interaction**	**0.0037**	**4.90E-03**	**CYSLTR2, GZMA, S1PR5**
**Metabolic pathways**	**0.0392**	**3.92E-02**	**PIGB, AMPD3, DHRS9, ENO1**

Bold means the pathway from KEGG

*FDR* false discovery rate

### Study 2: rapid transition program (RTP) cohort

Gene expression data from 218 patients were available at both baseline and convalescence. Baseline characteristics of patients are shown in Table 1. We used a FDR cutoff of 0.05 and determined which of these differentially expressed genes from study 1 (at a FDR of 0.05) were replicable in the Rapid Transition Program cohort. In study 1, there were 51 genes there were differentially expressed between the prednisone and control groups at a FDR < 0.05; of these, 32 genes were replicated at a FDR < 0.05 in study 2 (Additional file 5: Table S4). Enrichment analysis was also undertaken using these 32 genes, resulting in genes significantly enriched in 3 GO process. Similar to study 1, the highest regulated GO process was cytolysis (*p* = 1.57E-07), followed by cellular defense response (*p* = 0.0002), and immune response (*p* = 0.0005). KEGG pathway analysis resulted in 4 pathways including NK cell mediated cytotoxicity (*p* = 0.0001) (Additional file 6: Table S5).

**Fig. 3:**
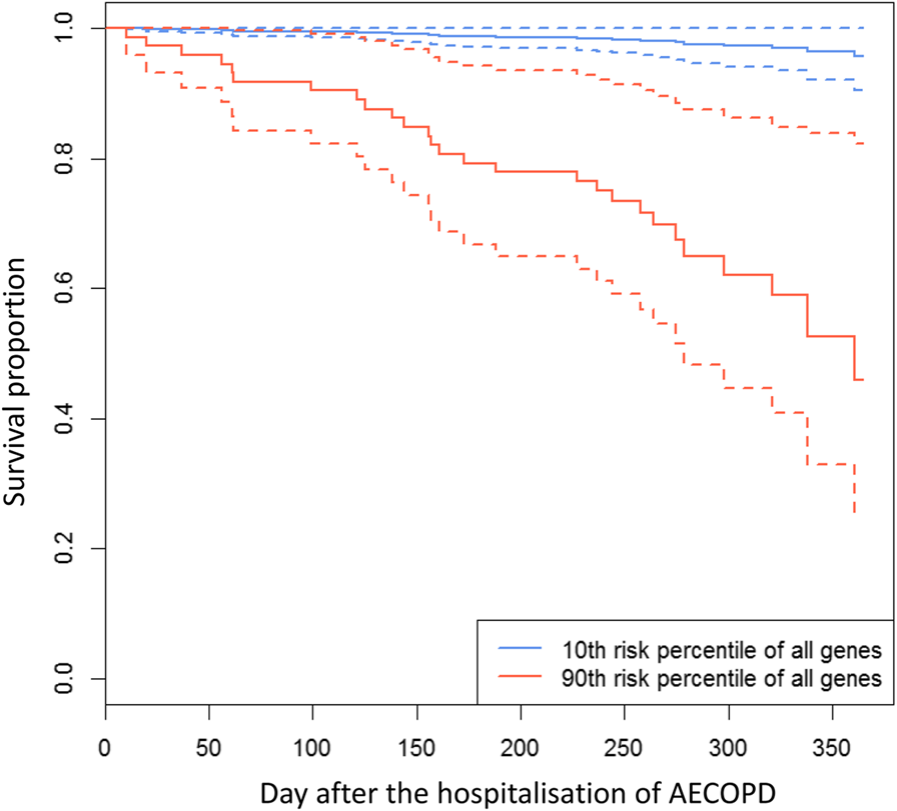
Theoretical survival curves based on gene signature of replicated 10 genes. 90th versus 10th risk percentile of 10 genes performed by fitting Cox proportional-hazards was shown. Broken lines represent the 95% confidence interval

Twenty-seven patients (12.4%) died within 1 year after hospitalisation related to AECOPD. Among the 32 replicated genes, changes in expression of 10 genes were significantly associated with 1-year mortality in a time-event-analysis (Table 4). Survival curves based on theoretical patients whose gene deltas lie at the 10th and 90th risk percentiles are shown in Fig. 3.

**Table 4:** Changes of gene expression level and their relationship to 1-year mortality in study 2

Gene	Gene name	Change in gene expression level with prednisone	1-year mortality	
			HR [95% CI]	*P*-value
CX3CR1	chemokine (C-X3-C motif) receptor 1	down	1.69 [1.01, 2.84]	0.0452
C1orf21	chromosome 1 open reading frame 21	down	1.69 [1.06, 2.68]	0.0276
KLRD1	killer cell lectin-like receptor subfamily D, member 1	down	1.67 [1.08, 2.60]	0.0215
S1PR5	sphingosine-1-phosphate receptor 5	down	1.65 [1.03, 2.62]	0.0364
ADGRG5	adhesion G protein-coupled receptor G5	down	1.63 [1.10, 2.43]	0.0160
PRF1	perforin 1 (pore forming protein)	down	1.63 [1.04, 2.57]	0.0346
TGFBR3	transforming growth factor beta receptor III	down	1.61 [1.05, 2.47]	0.0274
CYSLTR2	cysteinyl leukotriene receptor 2	down	1.57 [1.11, 2.22]	0.0108
NKG7	natural killer cell granule protein 7	down	1.43 [1.02, 2.02]	0.0393
DHRS9	dehydrogenase/reductase (SDR family) member 9	down	0.73 [0.54, 0.98]	0.0366

HR = decreased level of gene expression hazard rate / increased level of gene expression hazard rate from baseline to convalescence

*HR* hazard ratio, *95% CI* 95% confidence interval

## Discussion

Prednisone is used to treat moderate to severe COPD exacerbations but its effect on blood gene expression in COPD patients is largely unknown. Here, we showed in study 1 that short-term (4 day) prednisone therapy significantly modifies the expression of 51 genes in peripheral blood at a FDR < 0.05 (33 down-regulated and 18 up-regulated) and that these prednisone genes are significantly related to biological processes of immune systems including in the NK cell mediated cytotoxicity. Importantly, in study 2, we replicated 32 of 51 prednisone genes in a cohort of COPD patients hospitalised and treated with systemic corticosteroids for their AECOPD. Of these 32 genes, changes in the expression level of 10 genes were significantly associated with a mortality over 1 year.

The airways of COPD patients are characterized by the activation of inflammatory genes and infiltration of immune cells, which increases during acute exacerbations. The expression of inflammatory genes in the airway of COPD patients is controlled by pro-inflammatory transcription factors such as nuclear factor (NF)-κB [11]. Corticosteroids, on the other hand, repress the expression of inflammatory genes by inducing genes that encode for anti-inflammatory proteins such as inhibitor of NF-κB and by reversing histone acetylation of activated inflammatory genes via histone deacetylases (HDAC) 2 [12, 13].

Interestingly, in the differential analysis (between prednisone and control groups) in study 1, we found that a majority of genes that were significantly down-regulated at a FDR < 0.05 were those that were involved in NK cell function; KLRF1 (NKp80), ADGRG1, KLRD1 (CD94), NKG7, NCR1 (NKp46) and CX3CR1 or were cytotoxic mediators; GZMH, GZMA, GZMB, GNLY and PRF1. Furthermore, in enrichment analysis, prednisone therapy affected the pathway related to cellular immunity and cytolysis including NK cell mediated cytotoxicity. Similarly, in study 2, 11 genes modified by prednisone therapy during AECOPD were enriched in cellular immunity and NK cell mediated cytotoxicity pathways.

NK cells participate in innate immunity as a first line of defense against tumorigenesis or intracellular pathogens including viruses. This cytotoxic activity of NK cells is regulated by diverse combination of activating and inhibitory receptor-ligand interaction [14–20]. Granzyme is a family of serine proteases of which there are five known members in humans: A, B, H, K and M [21] and perforin is a pore-forming protein facilitating the entry of granzyme B into the target cell through a polyperforin pore [22]. Cytotoxic T lymphocytes and NK cells release inflammatory cytokines and cytolytic proteins to destroy target cells by inducing them to undergo apoptosis [18, 22, 23]. However, it is well-known that the predominant cells in the airways of COPD patients during AECOPD are neutrophils [15]. Our study did not find any significant effect of prednisone on genes involved in neutrophil-related pathways. This may explain why systemic corticosteroids are only modestly effective in improving patient outcomes in AECOPD.

In the survival analysis, KLRD1 and PRF1 genes, which are involved in the NK cell mediated cytotoxicity pathway was significantly related to 1-year mortality following AECOPD. We also found that CX3CR1 and S1PR5 genes, which encode receptors expressed on a variety of immune cells including NK cell and macrophages and regulate essential cellular processes such as cell proliferation, migration, adhesion and phagocytosis [24–26], were also significantly associated with 1-year mortality. CX3CR1 may play an important role in lung inflammation and is involved in emphysema pathogenesis related to cigarette smoke [25]. S1PR5, on the other hand, is expressed on alveolar macrophages of COPD patients and may regulate phagocytosis [24, 27]. Importantly, we showed that prednisone down-regulated the expression levels of all 10 genes, which, in turn, were associated with increased mortality. This raises the possibility that this 9 gene expression signature related to prednisone may be a biomarker of poor responsiveness or even harm related to systemic corticosteroids during AECOPD. However, these latter findings should be considered preliminary owing to the small sample size; additional validation is required in a larger cohort.

There were several limitations of this study. First, in study 1, we observed an up-regulation of NFKB2 which is counter to the known anti-inflammatory effects of prednisone (Additional file 3: Table S3). NFKB2 is a transcription factor which increases expression of genes, which promote inflammation. We postulate that the up-regulation in the level of expression of this gene related to prednisone therapy may be through a negative or indirect feedback mechanism by modulating the mitogen-activated protein kinase pathways or by activating inhibitors of NFKB [12]. Second, gene signature is tissue-specific, and thus our results cannot be generalised to lung tissue or airway epithelial cells. However, the strength of this study included the evaluation of whole blood which is easily accessible, enabling translation of these results to the clinic. Third, our gene signature related to short-term prednisone therapy was not validated at the protein level. Fourth, owing to the small sample size, we could not determine whether smoking modifies the effects of prednisone on gene expression. Previous studies have shown that smoking impairs therapeutic responses of patients to inhaled and systemic corticosteroids [28, 29]. In both study 1 and 2, we did not find any genes that were differentially expressed to patients’ smoking status at a FDR < 0.05.

## Conclusions

The current study has shown that short-term systemic corticosteroid therapy produces a distinct gene expression signature in blood of patients with COPD. A subset of these genes significantly relates to total mortality following COPD hospitalisation. Although our findings are preliminary and requires validation in larger studies, they raise the possibility that blood genomic signatures may be useful in identifying subgroups of patients who may experience benefits and others who may experience harm from systemic corticosteroid therapy.

## Additional files

Additional file 1: **Table S1.** Genes differentially expressed by prednisone at a FDR < 0.05 after adjusting for the total number of white blood cells and its differential cell count (day 3 versus day 1 in prednisone group). (DOCX 17 kb)

Additional file 2: **Table S2.** Genes differentially expressed by prednisone at a FDR < 0.05 after adjusting for the total number of white blood cells and its differential cell count (day 5 versus day 1 in prednisone group). (DOCX 16 kb)

Additional file 3: **Table S3.** Fifty-one genes differentially expressed by prednisone at a FDR < 0.05 in study 1. (DOCX 18 kb)

Additional file 4: **Figure S1** KEGG pathway map of NK cell mediated cytotoxicity. Rectangle and circle represent gene product including RNA and a compound, respectively. The blue boxes are hyperlinked to KEGG orthology entries and the red boxes represent genes which were significantly enriched in this pathway. Among genes which were responsive to short-term prednisone at a FDR < 0.05, KLRD1, PRF1, GZMB and NCR1 were significantly enriched in the pathway of NK cell mediated cytotoxicity at a FDR < 0.05. (PPTX 92 kb)

Additional file 5: **Table S4**. Genes replicated in the Rapid Transition Program (RTP) Cohort at a FDR < 0.05. (DOCX 16 kb)

Additional file 6: **Table S5**. Enrichment analysis: Replicated biological process and pathway in study 2. (DOCX 17 kb)

## Data Availability

The datasets used for the current study are available from the authors on reasonable request.

## Abbreviations

AECOPD: Acute exacerbation of chronic obstructive pulmonary disease
CBC: Complete blood count
COPD: Chronic obstructive pulmonary disease
FC: Fold change
FDR: False discovery rate
FEV1: Forced expiratory volume in 1 s
FVC: Forced vital capacity
GO: Gene ontology
GOLD: The Global Initiative for Chronic Obstructive Lung Disease
KEGG: Kyoto Encyclopedia of Genes and Genomes
RTP: Rapid Transition Program
