# Supplementary material for: Effect of short-term oral prednisone therapy on blood gene expression: a randomised controlled clinical trial
Source: Respir Res. 2019 Aug 5;20:176. doi: 10.1186/s12931-019-1147-2 (PMC6683462; doi:10.1186/s12931-019-1147-2)
Supplement: Supplementary file 1 — Table S1. Genes differentially expressed by prednisone at a FDR < 0.05 after adjusting for the total number of white blood cells and its differential cell count (day 3 versus day 1 in prednisone group). (DOCX 17 kb) [file 12931_2019_1147_MOESM1_ESM.docx]

**Table S1. Genes differentially expressed by prednisone at a FDR < 0.05 after adjusting for the total number of white blood cells and its differential cell count (day 3 versus day 1 in prednisone group).**

| Gene | Gene Name | *P*-value | FDR | FC | Direction |
| --- | --- | --- | --- | --- | --- |
| PIGB | phosphatidylinositol glycan anchor biosynthesis class B | 2.42E-06 | 3.22E-02 | 1.33 | down |
| NFKB2 | nuclear factor of kappa light polypeptide gene enhancer in B-cells 2 (p49/p100) | 4.51E-06 | 3.22E-02 | 1.21 | up |
| TPST1 | tyrosylprotein sulfotransferase 1 | 5.01E-06 | 3.22E-02 | 1.50 | down |
| PPP2R5C | protein phosphatase 2, regulatory subunit B, gamma | 9.38E-06 | 3.65E-02 | 1.16 | down |
| VAT1 | vesicle amine transport 1 | 9.48E-06 | 3.65E-02 | 1.16 | up |
| RARG | retinoic acid receptor, gamma | 1.7E-05 | 4.58E-02 | 1.16 | up |
| WLS | wntless Wnt ligand secretion mediator | 1.9E-05 | 4.58E-02 | 1.50 | down |
| GALC | galactosylceramidase | 2.13E-05 | 4.58E-02 | 1.18 | down |
| ECRP | ribonuclease, RNase A family, 2 (liver, eosinophil-derived neurotoxin) pseudogene | 2.43E-05 | 4.58E-02 | 1.58 | up |
| GZMB | granzyme B | 2.64E-05 | 4.58E-02 | 1.45 | down |
| POM121L9P | POM121 transmembrane nucleoporin-like 9, pseudogene | 2.69E-05 | 4.58E-02 | 1.28 | up |
| DHRS9 | dehydrogenase/reductase (SDR family) member 9 | 2.86E-05 | 4.58E-02 | 1.52 | down |

FDR, false discovery rate; FC, fold change.
