# Supplementary material for: Effect of short-term oral prednisone therapy on blood gene expression: a randomised controlled clinical trial
Source: Respir Res. 2019 Aug 5;20:176. doi: 10.1186/s12931-019-1147-2 (PMC6683462; doi:10.1186/s12931-019-1147-2)
Supplement: Supplementary file 2 — Table S2. Genes differentially expressed by prednisone at a FDR < 0.05 after adjusting for the total number of white blood cells and its differential cell count (day 5 versus day 1 in prednisone group). (DOCX 16 kb) [file 12931_2019_1147_MOESM2_ESM.docx]

**Table S2. Genes differentially expressed by prednisone at a FDR < 0.05 after adjusting for the total number of white blood cells and its differential cell count (day 5 versus day 1 in prednisone group).**

| Gene | Gene Name | *P*-value | FDR | FC | Direction |
| --- | --- | --- | --- | --- | --- |
| GZMB | granzyme B | 1.75E-06 | 1.54E-02 | 1.67 | down |
| PRF1 | perforin 1 (pore forming protein) | 2.49E-06 | 1.54E-02 | 1.58 | down |
| S1PR5 | sphingosine-1-phosphate receptor 5 | 2.91E-06 | 1.54E-02 | 1.50 | down |
| ADGRG1 | adhesion G protein-coupled receptor G1 | 3.19E-06 | 1.54E-02 | 1.79 | down |
| CEP78 | centrosomal protein 78kDa | 5.82E-06 | 1.58E-02 | 1.43 | down |
| SPON2 | spondin 2, extracellular matrix protein | 6.13E-06 | 1.58E-02 | 1.38 | down |
| C1orf21 | chromosome 1 open reading frame 21 | 6.49E-06 | 1.58E-02 | 1.69 | down |
| CX3CR1 | chemokine (C-X3-C motif) receptor 1 | 6.56E-06 | 1.58E-02 | 1.34 | down |
| SMAD7 | SMAD family member 7 | 8.00E-06 | 1.71E-02 | 1.29 | down |
| ID2 | inhibitor of DNA binding 2, dominant negative helix-loop-helix protein | 1.09E-05 | 2.09E-02 | 1.33 | down |
| VAT1 | vesicle amine transport 1 | 1.49E-05 | 2.49E-02 | 1.18 | up |
| NKG7 | natural killer cell granule protein 7 | 1.55E-05 | 2.49E-02 | 1.54 | down |
| TGFBR3 | transforming growth factor beta receptor III | 2.35E-05 | 2.92E-02 | 1.48 | down |
| KLRD1 | killer cell lectin-like receptor subfamily D, member 1 | 2.38E-05 | 2.92E-02 | 1.75 | down |
| PDGFD | platelet derived growth factor D | 2.38E-05 | 2.92E-02 | 1.65 | down |
| NPC1 | Niemann-Pick disease, type C1 | 2.43E-05 | 2.92E-02 | 1.32 | down |
| SYT2 | synaptotagmin II | 3.21E-05 | 3.64E-02 | 1.25 | down |

FDR, false discovery rate; FC, fold change.
