# Supplementary material for: Effect of short-term oral prednisone therapy on blood gene expression: a randomised controlled clinical trial
Source: Respir Res. 2019 Aug 5;20:176. doi: 10.1186/s12931-019-1147-2 (PMC6683462; doi:10.1186/s12931-019-1147-2)
Supplement: Supplementary file 3 — Table S3. Fifty-one genes differentially expressed by prednisone at a FDR < 0.05 in study 1. (DOCX 18 kb) [file 12931_2019_1147_MOESM3_ESM.docx]

**Table S3. 51 genes differentially expressed by prednisone at a FDR < 0.05 in study 1.**

| Gene | Gene Name | FC | *P*-value | FDR | Direction |
| --- | --- | --- | --- | --- | --- |
| KLRF1 | killer cell lectin-like receptor subfamily F, member 1 | 1.73 | 1.16E-06 | 2.48E-03 | down |
| GZMH | granzyme H | 1.64 | 1.84E-05 | 1.42E-02 | down |
| ADGRG1 | adhesion G protein-coupled receptor G1 | 1.58 | 8.37E-07 | 2.14E-03 | down |
| KLRD1 | killer cell lectin-like receptor subfamily D, member 1 | 1.54 | 5.04E-06 | 6.46E-03 | down |
| GZMA | granzyme A | 1.48 | 2.68E-05 | 1.90E-02 | down |
| DHRS9 | dehydrogenase/reductase (SDR family) member 9 | 1.48 | 1.31E-06 | 2.52E-03 | down |
| PDGFD | platelet derived growth factor D | 1.46 | 1.22E-05 | 1.13E-02 | down |
| GZMB | granzyme B | 1.46 | 3.08E-07 | 1.27E-03 | down |
| NKG7 | natural killer cell granule protein 7 | 1.42 | 1.88E-06 | 3.29E-03 | down |
| S1PR5 | sphingosine-1-phosphate receptor 5 | 1.40 | 3.30E-07 | 1.27E-03 | down |
| C1orf21 | chromosome 1 open reading frame 21 | 1.40 | 5.70E-05 | 2.74E-02 | down |
| GNLY | Granulysin | 1.38 | 4.84E-05 | 2.59E-02 | down |
| WLS | wntless Wnt ligand secretion mediator | 1.36 | 6.34E-05 | 2.91E-02 | down |
| SYTL2 | synaptotagmin-like 2 | 1.36 | 4.29E-05 | 2.43E-02 | down |
| PRF1 | perforin 1 (pore forming protein) | 1.35 | 3.83E-05 | 2.23E-02 | down |
| SLAMF7 | SLAM family member 7 | 1.33 | 2.53E-06 | 4.06E-03 | down |
| SPON2 | spondin 2, extracellular matrix protein | 1.33 | 1.96E-07 | 1.27E-03 | down |
| TGFBR3 | transforming growth factor beta receptor III | 1.28 | 1.05E-04 | 4.11E-02 | down |
| NCR1 | natural cytotoxicity triggering receptor 1 | 1.28 | 1.00E-04 | 4.09E-02 | down |
| PIGB | phosphatidylinositol glycan anchor biosynthesis class B | 1.28 | 8.89E-07 | 2.14E-03 | down |
| FAM174A | family with sequence similarity 174, member A | 1.27 | 3.55E-05 | 2.20E-02 | down |
| CEP78 | centrosomal protein 78kDa | 1.27 | 6.84E-05 | 3.06E-02 | down |
| ID2 | inhibitor of DNA binding 2, dominant negative helix-loop-helix protein | 1.27 | 2.27E-07 | 1.27E-03 | down |
| ABCB1 | ATP binding cassette subfamily B member 1 | 1.26 | 1.23E-05 | 1.13E-02 | down |
| OSBPL5 | oxysterol binding protein-like 5 | 1.24 | 1.02E-04 | 4.09E-02 | down |
| CYSLTR2 | cysteinyl leukotriene receptor 2 | 1.24 | 3.08E-06 | 4.23E-03 | down |
| SMPDL3A | sphingomyelin phosphodiesterase, acid-like 3A | 1.23 | 5.57E-05 | 2.74E-02 | down |
| MILR1 | mast cell immunoglobulin-like receptor 1 | 1.23 | 1.24E-04 | 4.78E-02 | up |
| USP28 | ubiquitin specific peptidase 28 | 1.23 | 3.83E-05 | 2.23E-02 | down |
| CX3CR1 | chemokine (C-X3-C motif) receptor 1 | 1.22 | 5.92E-05 | 2.78E-02 | down |
| ADGRG5 | adhesion G protein-coupled receptor G5 | 1.22 | 7.30E-06 | 8.78E-03 | down |
| TSPAN15 | tetraspanin 15 | 1.21 | 1.75E-05 | 1.42E-02 | up |
| STXBP1 | syntaxin binding protein 1 | 1.21 | 8.75E-05 | 3.66E-02 | up |
| ABI3 | ABI family, member 3 | 1.21 | 1.79E-05 | 1.42E-02 | down |
| ASPH | aspartate beta-hydroxylase | 1.21 | 9.89E-06 | 1.06E-02 | up |
| VAT1 | vesicle amine transport 1 | 1.21 | 3.11E-09 | 5.98E-05 | up |
| SYT2 | synaptotagmin II | 1.20 | 8.77E-06 | 9.93E-03 | down |
| NFKB2 | nuclear factor of kappa light polypeptide gene enhancer in B-cells 2 (p49/p100) | 1.20 | 2.84E-06 | 4.20E-03 | up |
| CD36 | CD36 molecule (thrombospondin receptor) | 1.19 | 3.02E-05 | 2.00E-02 | down |
| SYNE3 | spectrin repeat containing, nuclear envelope family member 3 | 1.19 | 7.34E-07 | 2.14E-03 | up |
| AMPD3 | adenosine monophosphate deaminase 3 | 1.18 | 2.76E-05 | 1.90E-02 | up |
| PER2 | period circadian clock 2 | 1.18 | 1.17E-05 | 1.13E-02 | up |
| MFGE8 | milk fat globule-EGF factor 8 protein | 1.17 | 3.25E-05 | 2.08E-02 | up |
| NR1D1 | nuclear receptor subfamily 1, group D, member 1 | 1.17 | 4.60E-05 | 2.53E-02 | up |
| ENO1 | enolase 1, (alpha) | 1.17 | 8.60E-05 | 3.66E-02 | up |
| ABCC2 | ATP binding cassette subfamily C member 2 | 1.14 | 1.27E-04 | 4.78E-02 | up |
| MAST4 | microtubule associated serine/threonine kinase family member 4 | 1.14 | 5.07E-05 | 2.64E-02 | up |
| PAG1 | phosphoprotein membrane anchor with glycosphingolipid microdomains 1 | 1.14 | 5.56E-05 | 2.74E-02 | up |
| RARG | retinoic acid receptor, gamma | 1.13 | 1.72E-05 | 1.42E-02 | up |
| RASA3 | RAS p21 protein activator 3 | 1.13 | 1.97E-05 | 1.46E-02 | up |
| IKBKE | inhibitor of kappa light polypeptide gene enhancer in B-cells, kinase epsilon | 1.12 | 7.44E-05 | 3.25E-02 | up |

FDR, false discovery rate; FC, fold change. The pooled data in prednisone group between day 3 and day 5 was compared with the pooled data in prednisone and control group at day 1. All gene expression data were adjusted for the total number of white blood cells and its differential cell count.
