# Supplementary figures and images for: Effect of short-term oral prednisone therapy on blood gene expression: a randomised controlled clinical trial
Source: Respir Res. 2019 Aug 5;20:176. doi: 10.1186/s12931-019-1147-2 (PMC6683462; doi:10.1186/s12931-019-1147-2)

## Slide 1
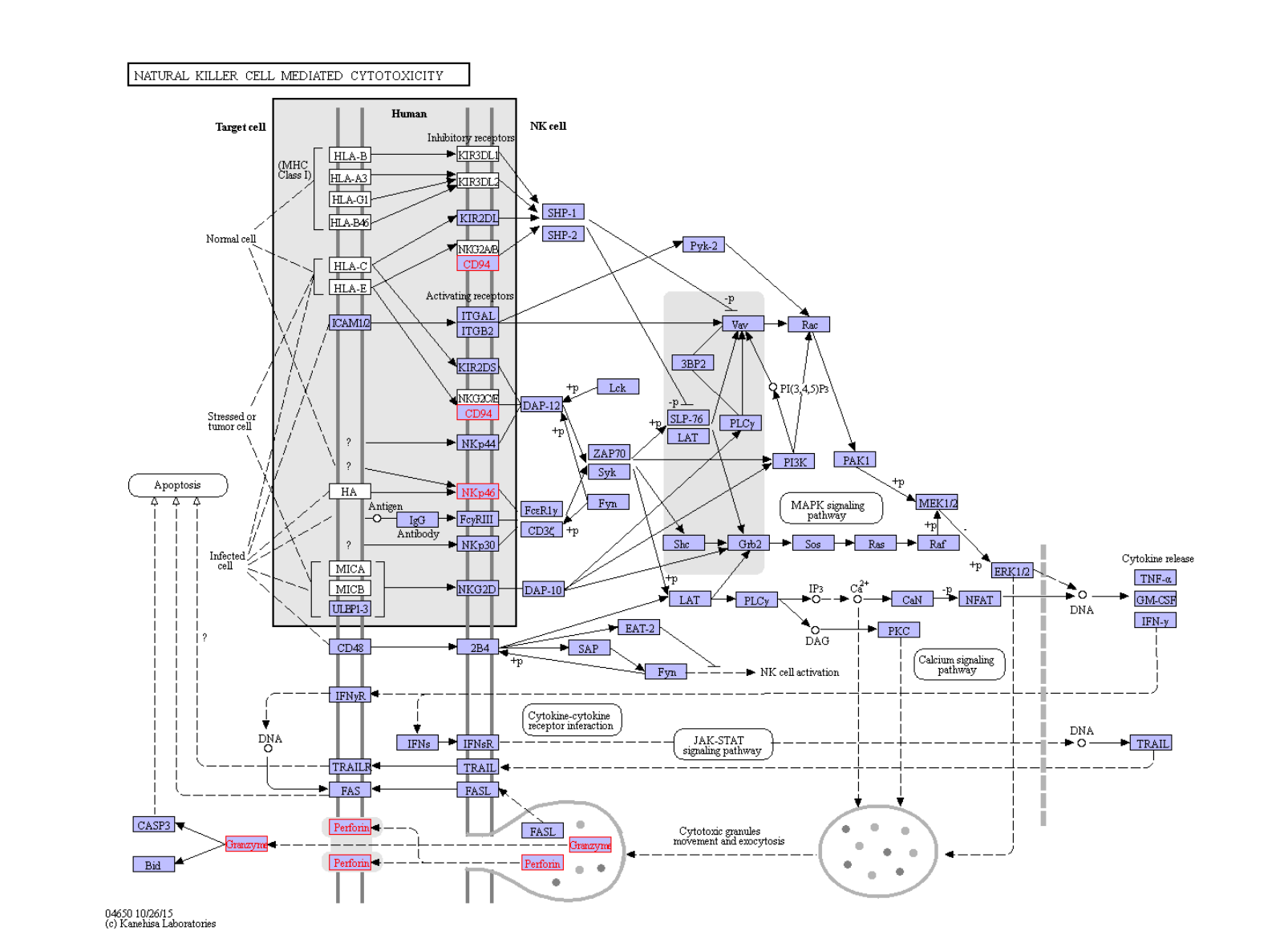

Supplement: Supplementary file 4 — Figure S1 KEGG pathway map of NK cell mediated cytotoxicity. Rectangle and circle represent gene product including RNA and a compound, respectively. The blue boxes are hyperlinked to KEGG orthology entries and the red boxes represent genes which were significantly enriched in this pathway. Among genes which were responsive to short-term prednisone at a FDR < 0.05, KLRD1, PRF1, GZMB and NCR1 were significantly enriched in the pathway of NK cell mediated cytotoxicity at a FDR < 0.05. (PPTX 92 kb) [file 12931_2019_1147_MOESM4_ESM.pptx]
