# Supplementary material for: Effect of short-term oral prednisone therapy on blood gene expression: a randomised controlled clinical trial
Source: Respir Res. 2019 Aug 5;20:176. doi: 10.1186/s12931-019-1147-2 (PMC6683462; doi:10.1186/s12931-019-1147-2)
Supplement: Supplementary file 5 — Table S4. Genes replicated in the Rapid Transition Program (RTP) Cohort at a FDR < 0.05. (DOCX 16 kb) [file 12931_2019_1147_MOESM5_ESM.docx]

**Table S4. Genes replicated in the Rapid Transition Program (RTP) Cohort at a FDR < 0.05.**

| Gene | Gene name | *P*-value | FDR | FC | Direction |
| --- | --- | --- | --- | --- | --- |
| ASPH | aspartate beta-hydroxylase | 3.28.E-11 | 1.67.E-09 | 1.40 | up |
| CYSLTR2 | cysteinyl leukotriene receptor 2 | 5.86.E-09 | 1.49.E-07 | 1.44 | down |
| GZMB | granzyme B | 1.16.E-06 | 1.97.E-05 | 1.28 | down |
| MFGE8 | milk fat globule-EGF factor 8 protein | 2.08.E-05 | 2.45.E-04 | 1.23 | up |
| PRF1 | perforin 1 (pore forming protein) | 2.40.E-05 | 2.45.E-04 | 1.24 | down |
| CX3CR1 | chemokine (C-X3-C motif) receptor 1 | 5.95.E-05 | 4.91.E-04 | 1.18 | down |
| VAT1 | vesicle amine transport 1 | 6.75.E-05 | 4.91.E-04 | 1.16 | up |
| ADGRG1 | adhesion G protein-coupled receptor G1 | 9.27.E-05 | 5.91.E-04 | 1.29 | down |
| TGFBR3 | transforming growth factor beta receptor III | 1.79.E-04 | 1.01.E-03 | 1.23 | down |
| GNLY | granulysin | 2.53.E-04 | 1.17.E-03 | 1.26 | down |
| C1orf21 | chromosome 1 open reading frame 21 | 2.72.E-04 | 1.17.E-03 | 1.22 | down |
| DHRS9 | dehydrogenase/reductase (SDR family) member 9 | 2.76.E-04 | 1.17.E-03 | 1.25 | down |
| KLRF1 | killer cell lectin-like receptor subfamily F, member 1 | 4.20.E-04 | 1.65.E-03 | 1.30 | down |
| GZMA | granzyme A | 5.75.E-04 | 2.10.E-03 | 1.20 | down |
| FAM174A | family with sequence similarity 174, member A | 6.48.E-04 | 2.20.E-03 | 1.16 | down |
| KLRD1 | killer cell lectin-like receptor subfamily D, member 1 | 9.37.E-04 | 2.99.E-03 | 1.21 | down |
| ENO1 | enolase 1, (alpha) | 1.13.E-03 | 3.39.E-03 | 1.12 | up |
| CEP78 | centrosomal protein 78kDa | 1.59.E-03 | 4.50.E-03 | 1.19 | down |
| PDGFD | platelet derived growth factor D | 1.83.E-03 | 4.92.E-03 | 1.18 | down |
| WLS | wntless Wnt ligand secretion mediator | 2.81.E-03 | 7.16.E-03 | 1.17 | down |
| PAG1 | phosphoprotein membrane anchor with glycosphingolipid microdomains 1 | 4.46.E-03 | 1.08.E-02 | 1.13 | up |
| MILR1 | mast cell immunoglobulin-like receptor 1 | 5.34.E-03 | 1.24.E-02 | 1.14 | up |
| AMPD3 | adenosine monophosphate deaminase 3 | 6.03.E-03 | 1.34.E-02 | 1.11 | up |
| ABCC2 | ATP binding cassette subfamily C member 2 | 7.29.E-03 | 1.55.E-02 | 1.14 | up |
| S1PR5 | sphingosine-1-phosphate receptor 5 | 7.69.E-03 | 1.57.E-02 | 1.15 | down |
| NKG7 | natural killer cell granule protein 7 | 8.43.E-03 | 1.65.E-02 | 1.19 | down |
| PIGB | phosphatidylinositol glycan anchor biosynthesis class B | 1.09.E-02 | 2.00.E-02 | 1.14 | down |
| GZMH | granzyme H | 1.10.E-02 | 2.00.E-02 | 1.17 | down |
| ABCB1 | ATP binding cassette subfamily B member 1 | 1.20.E-02 | 2.12.E-02 | 1.12 | down |
| SYTL2 | synaptotagmin-like 2 | 1.97.E-02 | 3.33.E-02 | 1.15 | down |
| ADGRG5 | adhesion G protein-coupled receptor G5 | 2.02.E-02 | 3.33.E-02 | 1.14 | down |
| SYNE3 | spectrin repeat containing, nuclear envelope family member 3 | 2.31.E-02 | 3.68.E-02 | 1.09 | up |

FDR, false discovery rate; FC, fold change.
