# Supplementary material for: Effect of short-term oral prednisone therapy on blood gene expression: a randomised controlled clinical trial
Source: Respir Res. 2019 Aug 5;20:176. doi: 10.1186/s12931-019-1147-2 (PMC6683462; doi:10.1186/s12931-019-1147-2)
Supplement: Supplementary file 6 — Table S5. Enrichment analysis: Replicated biological process and pathway in study 2. (DOCX 17 kb) [file 12931_2019_1147_MOESM6_ESM.docx]

**Table S5. Enrichment analysis: Replicated biological process and pathway in study2.**

| Biological process and pathway | *P-*value | FDR | Genes |
| --- | --- | --- | --- |
| cytolysis | 1.57E-07 | 2.26E-05 | GZMA, PRF1, GZMB, GZMH |
| **graft-versus-host disease** | **3.18E-06** | **1.27E-05** | **KLRD1, PRF1, GZMB** |
| **natural killer cell mediated cytotoxicity** | **0.0001** | **0.0002** | **KLRD1, PRF1, GZMB** |
| cellular defense response | 0.0002 | 1.44E-02 | PRF1, CX3CR1, GNLY |
| immune response | 0.0005 | 2.40E-02 | KLRD1, TGFBR3, GZMA, CX3CR1, MILR1, CYSLTR2, PRF1, PAG1 |
| **neuroactive ligand-receptor interaction** | **0.0009** | **0.0012** | **CYSLTR2,S1PR5, GZMA** |
| **metabolic pathways** | **0.0075** | **0.0075** | **PIGB, AMPD3, ENO1, DHRS9** |

Bold means the pathway from KEGG. FDR, false discovery rate.
